# Host Genetic Factors and Vaccine-Induced Immunity to Hepatitis B Virus Infection

**DOI:** 10.1371/journal.pone.0001898

**Published:** 2008-03-26

**Authors:** Branwen J. Hennig, Katherine Fielding, John Broxholme, Mathurin Diatta, Maimuna Mendy, Catrin Moore, Andrew J. Pollard, Pura Rayco-Solon, Giorgio Sirugo, Marianne A. van der Sande, Pauline Waight, Hilton C. Whittle, Syed M. Zaman, Adrian V. Hill, Andrew J. Hall

**Affiliations:** 1 London School of Hygiene & Tropical Medicine, London, United Kingdom; 2 Wellcome Trust Centre for Human Genetics, Oxford University, Oxford, United Kingdom; 3 Medical Research Council Laboratories, Banjul, The Gambia; 4 Centre for Clinical Vaccinology and Tropical Medicine, Oxford University, Oxford, United Kingdom; Université de Toulouse, France

## Abstract

**Background:**

Vaccination against hepatitis B virus infection (HBV) is safe and effective; however, vaccine-induced antibody level wanes over time. Peak vaccine-induced anti-HBs level is directly related to antibody decay, as well as risk of infection and persistent carriage despite vaccination. We investigated the role of host genetic factors in long-term immunity against HBV infection based on peak anti-HBs level and seroconversion to anti-HBc.

**Methods:**

We analyzed 715 SNP across 133 candidate genes in 662 infant vaccinees from The Gambia, assessing peak vaccine-induced anti-HBs level and core antibody (anti-HBc) status, whilst adjusting for covariates. A replication study comprised 43 SNPs in a further 393 individuals.

**Results:**

In our initial screen we found variation in IFNG, MAPK8, and IL10RA to affect peak anti-HBs level (GMTratio of <0.6 or >1.5 and P≤0.001) and lesser associations in other genes. Odds of core-conversion was associated with variation in CD163. A coding change in ITGAL (R719V) with likely functional relevance showed evidence of association with increased peak anti-HBs level in both screens (1st screen: s595_22 GMTratio 1.71, P = 0.013; 2nd screen: s595_22 GMTratio 2.15, P = 0.011).

**Conclusion:**

This is to our knowledge the largest study to date assessing genetic determinants of HBV vaccine-induced immunity. We report on associations with anti-HBs level, which is directly related to durability of antibody level and predictive of vaccine efficacy long-term. A coding change in ITGAL, which plays a central role in immune cell interaction, was shown to exert beneficial effects on induction of peak antibody level in response to HBV vaccination. Variation in this gene does not appear to have been studied in relation to immune responses to viral or vaccine challenges previously. Our findings suggest that genetic variation in loci other than the HLA region affect immunity induced by HBV vaccination.

## Introduction

Persistent hepatitis B virus (HBV) infection affects 350 million people world-wide, with highest endemicity in Sub-Saharan Africa and China. It is estimated that around half a million die every year from end stage complications of persistent infection [1]. Long-term studies in The Gambia aim to answer the question as to whether prevention of (early) HBV infection through infant vaccination reduces the incidence of persistent carriage and of subsequent hepatocellular carcinoma (HCC) in later life [2]–[9]. In The Gambia vaccination was introduced in the mid 1980s and has been demonstrated to be safe and effective, however, the level of HBV vaccine-induced antibody decays exponentially over time, and this is irrespectively of the population immunised [10]. The peak antibody level attained after vaccination is directly related to this uniform decay, as shown experimentally and through modelling [11], [12]. Hence we have used peak anti-HBs as a surrogate for durability since we can predict from it the antibody level at any time point afterwards. High peak anti-HBs level correlates directly with protection against infection and persistent carriage [9]. So whilst the anti-HBs level wanes with time since vaccination, the proportion of core-antibody (anti-HBc) positive individuals, i.e. those infected despite vaccination, increases and was shown to be >15% in the most recent surveys carried out (with up to 19 years of follow-up) [9], [12]. This happens at a time when the risk of exposure is increasing due to the onset of sexual activity.

Little is known about host genetic determinants of immunity induced by infant HBV vaccination *per se*, and even less about the role of genetic variation in determining the duration of vaccine-induced immunity. Family and twin studies have established that genetic variability influences vaccine immunogenicity [13]–[15]. Höhler *et al* concluded from a twin study assessing the heritability of HBsAg vaccine response that genetic factors have a strong influence, with variation in the MHC playing an important role, but suggesting that more than half the heritability is determined by non-MHC genes [16]. In The Gambia Newport and colleagues assessed the heritability of immune responses to several infant vaccines in twins and showed that the genetic contribution to antibody responses varied depending on the vaccine; for anti-HBs response the observed heritability was relatively high (77%) [17].

A number of studies have linked HLA class I, II and III variation to non-response or low response to hepatitis B vaccine [18]–[28]. The most consistent findings regarding high response are associations with DRB1*01 (DR1), DRB1*11 (DR5), DRB1*15 (DR2), DQB1*0501, DPB1*0401; non-response has been shown repeatedly to correlate with DRB1*03, DRB1*07, DQB1*02, DPB1*1101 [29], [30]. However, very little indeed is published on HBV vaccine-induced immunogenicity regarding variation in non-HLA genes [31]. Most of these studies are hampered by small sample sizes and a limited number of markers/genes screened, so we only have a snapshot understanding of what genetic factors are implicated in the control of HBV vaccine immunogenicity. Furthermore, different populations have been studied making comparisons difficult, and virtually none of these associations have been confirmed. Finally, although we know that peak anti-HBs level predicts long-term vaccine efficacy, what genetic factors may influence duration of protection has hardly been investigated and we do not know whether the genetic control of short-term immune responses as measured in most studies translate into durable immunity – it is likely that different genetic mechanisms underlie the control of immune memory.

This current study is, to our knowledge, the first project assessing this large a number of (non-HLA based) candidate genes and their possible effect on HBV vaccine-induced immunity. We obtained results on a total of 715 single nucleotide polymorphisms (SNPs) across 133 genes, and tested for association with peak anti-HBs level (directly correlated with durability of anti-HBs response, i.e. long-term vaccine efficacy, protection against infection and persistent carriage) and anti-HBc status (indication of infection despite vaccination) in a study sample of 662 infant vaccinees from The Gambia who have been followed-up for close to two decades. We report here on the findings from this study, together with a replication study comprising 43 SNPs in 393 further individuals from the same population.

## Materials and Methods

### Study participants

Recruitment was carried out in 2003 in the West Kiang region in The Gambia as part of the survey to determine HBV vaccine efficacy 19 years post vaccination, and in particular to evaluate of the magnitude and duration of protective antibody responses induced by infant HBV vaccination [9]. Findings from earlier studies in the same region (1985, 1989, 1993, and 1998) including information on demographics, medical background and epidemiological data have been published previously [2], [5]–[9]. Briefly, children born within the area covered by the MRC Unit in Keneba (West-Kiang, The Gambia) vaccinated within the HBV vaccination programme were considered eligible for the present study. The recruitment was restricted to non-infected individuals, only confirmed non-immune children <5 years old had been recruited prior to 1985 and from there onwards all study participants were vaccinated at birth, i.e. are deemed anti-HBc negative (perinatal transmission of HBV infection is very rare in this population[32]).

This genetic study was carried out in two stages: The first screen study participants comprised all individuals identified as anti-HBc positive on at least one survey (i.e. ever positive) and, where possible, two randomly selected age-group matched consistently anti-HBc negative individuals. All other individuals, who by definition were anti-HBc negative, were included in the second stage, which was designed with the aim to replicate the ‘top’ results from the first screen. Exclusion criteria were: no information on peak anti-HBs level or date, peak anti-HBs measurement time less than one month after the last vaccination (anti-HBs is thought to peak at 1 month after the last dose), no information on number of doses of vaccine and age >5 yrs at time of last vaccination. Study participants originated from three main villages Keneba, Manduar and Kantonkunda and a small number of individuals from other villages or visitors to the region, all of whom had been included in the vaccine programme. Vaccination groups up to 2002 reflect type of vaccine, administration route and time period during which a regime was used (see Table 1); accordingly there is a direct relationship between vaccine group and date of birth (i.e. age of vaccinee). The number of individual belonging to vaccine group 6 was small (n = 2) and these were thus excluded from the analysis. The number of vaccine doses received was grouped into ≤3 (containing a small number of two and one dose recipients) versus 4 doses. An overview of selection/exclusion criteria is given in Supplementary Figure S1.

**Table 1:** Frequency distribution of outcome measures and covariates in 1^st^ and 2^nd^ screen sample sets with corresponding geometric mean peak anti-HBs titer (GMT)

		1st screen participants (n = 662)					2nd screen participants (n = 393)				
	Grouping	N	*%*	GMT	95%CI		N	*%*	GMT	95%CI	
**Age group [years]**	0–5	51	*7.7*	180	106	306	118	*30.0*	200	141	284
	5–10	120	*18.1*	436	289	658	175	*44.5*	900	660	1226
	10–15	184	*27.8*	1957	1523	2515	96	*24.4*	3665	2631	5106
	15–20	227	*34.3*	2195	1613	2986	4	*1.0*	5566	884	35039
	>20	80	*12.1*	411	262	646	n/a				
**Gender**	male	317	*47.9*	1009	783	1300	207	*52.7*	703	519	951
	female	345	*52.1*	1126	885	1432	186	*47.3*	978	712	1344
**Village**	Keneba	368	*55.6*	1121	886	1418	186	*47.3*	891	646	1228
	Manduar	150	*22.7*	1091	750	1587	81	*20.6*	957	596	1537
	Kantonkunda+others	144	*21.8*	924	643	1329	126	*32.1*	662	448	980
**Vaccination group**	1 (1984/1985)	54	*8.2*	987	672	1449	n/a				
	2 (1984/1985)	43	*6.5*	443	279	705	n/a				
	3 (1984/1985)	45	*6.8*	163	92	290	excluded (n = 3)				
	4 (1984–1992)	294	*44.4*	3263	2557	4165	80	*20.4*	4355	3068	6182
	5 (1992–1993)	62	*9.4*	1049	705	1562	39	*9.9*	1757	1039	2973
	6 (1992–1993)						excluded (n = 2)				
	7 (1993–2001)	164	*24.9*	314	224	442	274	*69.7*	453	351	586
**Number of doses**	≤3	273	*41.2*	567	432	744	118	*30.0*	266	184	384
	4	389	*58.8*	1666	1342	2069	275	*70.0*	1334	1039	1713
**Ethnicity**	Mandinka	636	*96.1*	1066	892	1274	367	*93.4*	823	656	1032
	Other	26	*3.9*	1127	493	2572	26	*6.6*	808	319	2043
**Vaccine response**	non-responder (<10IU/ml)	32	*4.8*				15	*3.8*			
	responder (≥10IU/ml)	630	*95.2*	1403	1202	1638	378	*96.2*	1006	822	1231
**Anti-HBc status**	always negative	462	*69.8*	1560	1280	1900	n/a				
	consistently positive	132	*19.9*	298	197	452	n/a				
	reconverter	61	*9.2*	1021	626	1666	n/a				
	variable	7	*1.1*	629	99	3984	n/a				

*Key:* Vaccination groups: 1) HB-Vax 3× 20 µg im; 2) HB-Vax 1× 20 µg im+2× 2 µg id; 3) HB-Vax 3× 2 µg id; 4) HB-Vax 4× 10ug im; 5) Recombivax 3× 5 µg im; 6) EnergixB 3× 10 µg im; 7) Hepacine 4× (later 3×) 2.5 µg im [9]. 95%CI: 95% confidence interval.

*Notes on 1^st^ screen:* The ‘Kantonkunda+others’ village group includes 33 individuals from other villages or visitors to the region. No individuals in vaccine group 6 were present. The number of individuals with less than 3 doses includes three participants with only two doses of HBV vaccine.

*Notes on 2^nd^ screen:* As there were only four individuals aged >15 years, these were grouped with those aged 10–15 years. The ‘Kantonkunda+others’’ village group contains 44 ‘others’. There were no individuals from vaccination groups 1 and 2; vaccine groups 3 and 6 were excluded from the analysis as they contained less than five individuals in each. The number of individuals with less than 3 doses included a total of one individual with one dose, and six participants with two doses of HBV vaccine.

We studied two outcome measures in the 1^st^ screen (i) peak anti-HBs level as quantitative variable and (ii) anti-HBs status as binary variable, and for the 2^nd^ screen peak anti-HBs level only. Additional screen specific exclusion of samples and covariate frequency distribution with corresponding geometric mean titer (GMT) of peak anti-HBs for each of the two sample sets are described below and are summarized in Table 1.

This project was approved by the joint Gambia Government/MRC Ethics Committee, as well as LSHTM and Oxford University (OXTREC) Ethics Committees. All subjects and/or legal guardians provided written, informed consent.

#### 1^st^ screen study participants

The total available sample for the first screen with complete data on covariates was 662, these were included in the assessment of peak anti-HBs level. The number of individuals included in the analysis of anti-HBc status was reduced to 594, because we decided to confine the analysis to a clear-cut binary outcome of consistently anti-HBc positive versus consistently negative individuals. Thus, we excluded individuals who were reconverters (n = 61; 9.3%), i.e. those shown to have lost core-antibody positivity over the course of follow-up, and those who presented with variable anti-HBc status over time (n = 7). The group of individuals with less than 3 doses of vaccine included two individuals with two doses only. The mean age of the whole 1^st^ screen sample was 13.4 years (range 1.2 to 22.9 years) and consisted of 377 sibships with up to seven siblings, 27.1% of these sibships comprised just one individual; relatedness was accounted for in the analysis.

#### 2^nd^ screen study participants

The sample available for analysis for the 2^nd^ screen was 393 individuals with complete data on covariates, the mean age was 7.4 years (range 1.1 to 16.2 years). These individuals tended to be younger and all were consistently anti-HBc negative, due to the initial selection procedure applied for the 1^st^ screen (see above). The group of individuals with less than 3 doses of vaccine included six with two doses and one with one dose. The 2^nd^ screen sample consisted of 277 sibships with up to five siblings, 42.2% of these contained only one individual. There were no individuals representative of vaccine group 1 and 2 in this sample (due to the younger mean age) and because groups 3 and 6 consisted of very few individuals, the five participants belonging to these vaccine groups were excluded.

### Vaccinations and serological assessment

Vaccination types and regimes changed over time (as shown in Table 1). The median measurement time between last vaccination and peak antibody level assessment was 9 weeks (range 5.0 to 57.2 weeks). At each survey time point, concentrations of core antibody (anti-HBc) and, if found to be positive, hepatitis B surface antigen (HBsAg) and HBeAg were assessed, as described previously [9].

Infection was defined as the presence of anti-HBc with at least 30% inhibition, as stipulated by the manufacturer (Sorin Biochemica or DiaSorin). If infection was found only during a single follow-up time point and was not confirmed subsequently, the person was considered to have had a transient infection. Carriage was defined as the detection of HBsAg on two separate occasions at least six months apart. Primary vaccine failure was defined as a peak anti-HBs response of <10 mIU/mL; a responder was defined as a vaccinee with a peak anti-HBs response of ≥10 mIU/mL. The proportion of HBsAg positive (n = 21), anti-HBeAg positive (n = 7) and persistent carriers (n = 7) in the total sample was too small for a subgroup analysis of these parameters.

### Genetic analysis

DNA extraction from whole blood or PBMCs was carried out using a standard salting-out method [33]. Genotyping assays for the initial screen were run on the Illumina BeadArray platform (www.illumina.com). Our candidate gene selection criteria were based on literature searches, previous reported genetic associations with vaccine-induced antibody level (in particular with anti-HBs), possible biological implication in immune-regulatory processes, inclusion of gene families and coverage of regulatory pathways. The selection of 768 single nucleotide polymorphism (SNPs) for genotyping was based on HapMap frequency data, validation, anticipated success rate on the Illumina platform, gene size, distribution across loci, enrichment in coding changes, inclusion of sequence 500 bp up- and down-stream of each gene. Just over 95% of our candidate polymorphisms were SNPs genotyped in both Yoruba (YRI) and Caucasians (CEU) as part of the HapMap project (http://www.hapmap.org/), many of these variants are tag SNPs in either or both of these populations.

Linkage disequilibrium (LD) and haplotype blocks were assessed using the Haploview programme (http://www.broad.mit.edu/mpg/haploview/) applying default parameters according to Gabriel *et al* [34]. To account for markers with low minor allele frequency (MAF) we employed r^2^ as measure of LD throughout and considered an r^2^ value of 0 to 0.5 as low, 0.5 to 0.75 as intermediate and >0.75 as strong LD. The FASTSNP programme (http://fastsnp.ibms.sinica.edu.tw/pages/input_CandidateGeneSearch.jsp) was employed to establish whether SNPs were of functional relevance [35].

The 2^nd^ screen genotyping was carried out using the Sequenom platform (iPlex and hME; http://www.sequenom.de/). The aim was to replicate the results from the first screen by concentrating on the ‘top’ markers in the remainder of the sample set.

### Statistical analysis

Validated demographic and serological data was available as an Access 2000 database from the MRC's long-term hepatitis B vaccination programme [9]. All statistical analysis was conducted using Stata software (version 9; StataCorp) and employed forward regression models to determine the role of genetic markers on outcomes, with inclusion of individual SNPs into the (multi-marker) gene/locus models according to LD structure. No adjustment for multiple comparisons was made. Almost 95% of individuals were Mandinka, ethnicity was thus not considered informative enough for stratification of the study cohort by ethnic groups.

This society is polygamous, thus resulting in a complex pedigree structure, e.g. presence of a large proportion of half-sibs. Consequently, clustering by sibship was determined by maternal ID in order to account for relatedness. For polymorphisms with a low MAF heterozygotes and homozygotes were grouped for the analysis if there were less than 10 individuals in the latter genotype category by outcome measure. Genetic variation was entered in the analysis as categorical genotype data with 11 representing ancestral homozygotes, 12 representing heterozygotes and 22 representing variant homozygotes. Two outcome measures were assessed: (i) peak anti-HBs level and (ii) anti-HBc status.

#### i) Peak anti-HBs

From the peak anti-HBs level we can predict the persistence of antibody level at any time point afterwards, peak anti-HBs is also a predictor of risk of infection and persistent carriage. Peak anti-HBs was not normally distributed and thus log-transformed for the analysis. Multiple linear regression, including robust standard errors to account for relatedness by sibship, was used to identify covariates associated with log peak anti-HBs level. The model included as covariates the logarithm of measurement time (i.e. duration of time between last vaccination and peak anti-HBs measurement to account for the exponential decay of anti-HBs over time), vaccine group (i.e. type, regime, administration route, year), number of doses (≤3 versus 4), age group at recruitment (5 year intervals), village (Keneba, Manduar, Kantonkunda+others), gender and relatedness. Results are summarized as the ratio of the geometric mean titer (GMT), associated 95% confidence intervals (CI) and P-values.

#### ii) Anti-HBc status

Consistent core antibody positive individuals were defined as those who had been identified as anti-HBc positive at any time point and remained positive at subsequent surveys and compared to subjects who were consistently negative for anti-HBc over the course of time; reconverters and variables were excluded (see above). Multiple conditional logistic regression, including robust standard errors to account for relatedness by sibship, was used to identify covariates associated with being core antibody positive. The model for anti-HBc status accounted for peak anti-HBs level (as surrogate measure for vaccine group, anti-HBs measurement time, number of vaccine doses), age group at recruitment (5 year intervals), village (Keneba, Manduar, Kantonkunda+others), and gender as covariates. Results are reported as odds ratios (OR), with associated 95% CI and P-values.

The statistical analysis for the 2^nd^ screen was carried out in an identical manner to that of the 1^st^ screen for anti-HBs level as the outcome. The distribution of covariates for the 2^nd^ screen was slightly different in terms of age and vaccine group distribution (see below).

## Results

### Genetic analysis of 1^st^ screen data

Overall 739/768 SNP assays produced good quality data (failure rate 3.8%). Twenty SNPs were monomorphic or had a MAF of <1% and were excluded from the analysis. The remaining SNPs were distributed across 133 candidate genes with a varying number of markers per gene (shown in supplementary Table S1), covering all chromosomes except 15 and 18. The average genotyping success rate across all markers was 99.5% (ranging from 71.2–100.0%). Four SNPs had a failure rate >10% and were excluded leaving a total of 715 SNPs for analysis.

Hardy-Weinberg equilibrium (HWE) calculation was based on one randomly selected individual from each sibship. Of the 715 SNPs available for the statistical analysis 40 (5.6%) were shown to diverge from HWE (P range 1.5E-27 to 0.5). At the P<0.05 level we expect to find approximately 36 SNPs to be out of HWE, the observed number of markers diverging from HWE is therefore comparable to the expected number. There was no obvious clustering of SNPs out of HWE, or a trend for excess presence of one particular genotype across these polymorphisms. Markers not in HWE are only discussed further if a positive association with either outcome measure was found.

### Statistical analysis of 1^st^ screen data

#### i) peak anti-HBs level

##### Basic model for peak anti-HBs level for 1^st^ screen

In all models, including the basic model (i.e. excluding genetic factors, see Table 2A), a strong effect of vaccine group and measurement time was seen on peak anti-HBs as quantitative outcome measure. Overall, the adjusted GMT was between 60–94% lower in all vaccination groups compared to the reference group 4 (P≤0.001). This was expected and previously described as part of the HBV vaccine efficacy studies in this population [9]. The adjusted correlation coefficient for the decline of anti-HBs level with time since last vaccination (measurement time) was −0.99 (95%CI: −1.51 to −0.47). No effect was seen with number of vaccine doses (≤3 vs 4) upon inclusion or exclusion of the few individuals with only two doses of vaccine (data not shown). Village seemed to show a trend towards association, with individuals born in Kantonkunda presenting with a slightly decreased vaccine-induced antibody level. This result did not change upon exclusion of the 33 individuals from other villages or visitors to the region, who had been grouped with Kantonkunda for the analysis (data not shown). Neither gender nor age at recruitment (range 1.2 to 22.9 years) showed an effect on peak anti-HBs level.

**Table 2:** Basic models using outcome measures peak anti-HBs level for 1^st^ and 2^nd^ screen and anti-HBc status for 1^st^ screen only

Outcome: Peak anti-HBs level		1^st^ screen				2^nd^ screen			
Covariates [baseline]	Grouping	Adjusted GMTratio	95%CI		P	Adjusted GMTratio	95%CI		P
**ln(measure time)** a		**−0.99 ** a	−1.51	−0.47	**2.35E-04**	**−1.25**	−1.81	−0.69	**1.78E-05**
**Vaccination group ** b [4]	1	**0.40**	0.19	0.86	**0.018**	n/a			
	2	**0.18**	0.08	0.39	**1.40E-05**	n/a			
	3	**0.06**	0.03	0.15	**1.93E-10**	excluded (n = 3)			
	5	**0.42**	0.25	0.7	**0.001**	**0.47**	0.23	0.97	**0.041**
	7	**0.15**	0.04	0.52	**0.003**	**0.30**	0.10	0.88	**0.028**
**Number of doses** [3]	4	1.51	0.8	2.87	0.204	0.68	0.36	1.28	0.227
**Age group in years ^c^** [0–5]	5–10	0.63	0.27	1.45	0.278	1.59	0.71	3.56	0.262
	10–15	0.52	0.13	2.05	0.345	2.05	0.58	7.26	0.264
	15–20	1.00	0.25	4.01	0.999	2.78	0.47	16.26	0.256
	>20	0.76	0.17	3.33	0.713	Combined with age group 15–20			
**Gender** [male]	female	0.9	0.67	1.22	0.494	1.18	0.82	1.7	0.374
**Village** [Keneba]	Manudar	0.83	0.55	1.26	0.386	0.99	0.62	1.58	0.981
	Kantonkunda + others	**0.62**	0.42	0.94	**0.023**	0.86	0.55	1.36	0.528

Outcome: Anti-HBc status		1^st^ screen			
Covariates [baseline]	Grouping	Adjusted OR	95%CI		P
**ln(anti-HBs)**		**0.7**	0.63	0.77	4.83E-13
**Age group in years** [0–5]	5–10	1.57	0.6	4.08	0.355
	10–15	2.05	0.81	5.23	0.131
	15–20	3.58	1.42	9.01	0.007
	>20	2.88	1.06	7.83	0.038
**Village** [Keneba]	Manduar	1.36	0.81	2.28	0.249
	Kantonkunda + others	**1.66**	1.02	2.7	**0.04**
**Gender** [male]	female	0.68	0.45	1.01	0.057

*Key:* GMTratio: Geometric mean peak anti-HBs titer relative to baseline by covariate (i.e. age group 0–5, number of doses ≤3, vaccine group 4, village Keneba, gender male). ln(measurement time): time between last vaccination and peak anti-HBs measurement in weeks. 95%CI: 95% Confidence intervals. OR: odds ratio.

a The decline of anti-HBs level with time since last vaccination (measurement time) is expressed as the decrease in peak anti-HBs level for a unit increase in log(measurement time) with corresponding P-value and 95%CI, adjusted for all covariates.

b *Notes on 2nd screen:* Age >15 yrs grouped together; vaccine groups 3, and 6 excluded, groups 1 and 2 not present. For further details on sub-grouping and exclusion of subcategories see Table 1 and text. Anti-HBs level had a dose-dependent effect on risk of core-conversion; re-converters and variable positives (n = 61 and n = 7, respectively) were excluded from the analysis.

##### Adjusted genetic models for peak anti-HBs for 1^st^ screen

Out of a total of 133 screened genes we identified host genetic variation in three candidate genes to affect peak vaccine-induced antibody level using a cut-off of size of effect (ratio of GMT) of <0.6 or >1.5 and P≤0.001 in the multi-marker models adjusted for covariates, namely: MAPK8, IL10RA, and IFNG (see Table 3). For these three genes the multi-marker models indicated a similar modification of GMT, but with reduced significance compared to single SNP models (data not shown).

**Table 3:** Outcome anti-HBs level and anti-HBc status adjusted for covariates for 1^st^ screen and replicated data

1^st^ Screen results														
Gene	dbSNP	Genotype	Outcome:				Genotype	Outcome:				Genotype frequencies		
			peak anti-HBs level					anti-HBc status				11	12	22
			GMTratio	P	95%CI			OR	P	95%CI		*%*	*%*	*%*
**CD58**	rs1414275	s062_12	**0.49**	**1.75E-05**	0.36	0.68	s062_12	1.26	0.374	0.75	2.11	239	303	109
		s062_22	**0.43**	**2.88E-04**	0.27	0.68	s062_22	**1.71**	0.090	0.92	3.19	*36.7*	*46.5*	*16.7*
	rs1016140	s064gp_33	**0.39**	**4.00E-06**	0.26	0.58	s064gp_33	1.36	0.213	0.84	2.21	528	116	4
												*81.5*	*17.9*	*0.6*
**MAPK8**	rs3827680	s419_12	0.66	0.015	0.48	0.92	s419_12	0.65	0.065	0.41	1.03	210	327	113
		s419_22	**0.42**	**3.16E-04**	0.26	0.67	s419_22	0.89	0.709	0.48	1.65	*32.3*	*50.3*	*17.4*
	rs10857565	s420gp_33	**0.57**	0.025	0.35	0.93	s420gp_33	1.22	0.521	0.67	2.23	560	91	1
												*85.9*	*14.0*	*0.6*
**IL10RA**	rs2508450	s481_12	1.75	0.001	1.27	2.41	s481_12	1.01	0.953	0.65	1.59	369	243	40
		s481_22	1.46	0.272	0.74	2.86	s481_22	**1.66**	0.237	0.72	3.82	*56.6*	*37.3*	*6.1*
	rs2229113	s483_12	0.93	0.691	0.67	1.31	s483gp_33	1.21	0.395	0.78	1.90	483	151	16
		s483_22	0.82	0.603	0.38	1.75						*74.3*	*23.2*	*2.5*
**CD163 ** a	rs6488340	s503_12	1.06	0.778	0.72	1.54	s503_12	1.80	0.028	1.07	3.02	221	303	126
		s503_22	1.03	0.885	0.67	1.60	s503_22	**2.17**	0.011	1.19	3.95	*34.0*	*46.6*	*19.4*
	rs4883263	s504_12	1.06	0.740	0.74	1.52	s504_12	1.01	0.956	0.65	1.57	220	301	128
		s504_22	1.02	0.917	0.65	1.63	s504_22	0.87	0.661	0.45	1.65	*33.9*	*46.4*	*19.7*
**IFNG**	rs2069727	s523gp_33	**1.97**	**1.30E-04**	1.40	2.78	s523gp_33	0.63	0.149	0.34	1.18	544	104	4
												*83.4*	*15.9*	*0.6*

Replicated data (1^st^ and 2^nd^ screen)														
Gene	dbSNP	Genotype	Outcome peak anti-HBs level					Outcome peak anti-HBs level				Genotype frequencies(1^st^ screen) b		
			1^st^ screen					2^nd^ screen				11	12	22
**ITGAL**	rs4243232	s594_12	1.01	0.938	0.71	1.45	s594_12	0.84	0.440	0.54	1.30	370	245	36
		s594_22	**1.77**	0.029	1.06	2.95	s594_22	1.70	0.178	0.78	3.68	*56.8*	*37.6*	*5.5*
	rs2230433	s595_12	1.11	0.588	0.76	1.63	s595_12	1.19	0.440	0.76	1.86	254	274	122
		s595_22	**1.71**	0.013	1.12	2.62	s595_22	**2.15**	**0.011**	**1.19**	**3.88**	*39.1*	*42.2*	*18.8*

*Key:* GMTratio: Geometric mean peak anti-HBs titer adjusted for covariates. OR: odds ratio adjusted for covariates. 95%CI: 95% Confidence intervals.

The results shown represent single SNP models adjusted for covariates with associations of GMTratio/OR of <0.6 or >1.5 and P≤0.001 level (for other markers taken forward to 2^nd^ screen see Supplementary Table S2). The genotype designation are 1 for ancestral and 2 for variant allele; _33 indicates the grouping of homozygote variants (22) and heterozygotes (12). Multi-marker models affecting peak anti-HBs level at these cut-off values were MAPK8, IL10RA and IFNG. CD163 was correlated with anti-HBc status in the multi-SNP analysis. For all other genes the multi-marker models indicated a comparable modification of GMT, but with reduced significance compared to single SNP models (data not shown), except for the CD163 anti-HBc model (see below). The association of ITGAL s595 (rs2230433) with anti-HBs level was replicated. SNPs s321 and s574 were out of HWE at the P<0.05 level.

a CD163 multi-SNP model for anti-HBc: s503_12 GMTratio 3.89, P = 0.006, 95%CI 1.5–10.1; s503_22 GMTratio 7.24, P = 2.31E-04, 95%CI 2.5–20.75; s504_12 GMTratio 1.74, P = 0.091, 95%CI 0.9–3.30; s504_22 GMTratio 4.53, P = 0.009, 95%CI 1.5–14.1

b Genotypes 2^nd^ screen: s594_11 = 170 s594_12 = 113 s594_22 = 24; s595_11 = 146 s595_12 = 165 s595_22 = 50.

##### IFNG

Carriage of the variant allele for s523, which is located just outside the 3′UTR, was associated with a two times increase in anti-HBs level (P = 0.0001), FastSNP predicts this polymorphism to be of functional relevance. The two screened markers in IFNG showed low pairwise LD (r^2^ = 0.27), and the associated SNP (s523) was not in LD with markers in neighbouring genes, i.e. IRAK3 upstream, or IL26 and IL22 downstream. We therefore suspect this to be an independent association within the IFNG locus.

##### MAPK8 (JNK1)

A SNPs in MAPK8, s419 (intronic), demonstrated a linear trend of carriage of the variant allele with 40–60% reduction in anti-HBs level (P = 0.0003). A second marker, s421, which is in almost complete LD with s419 (r^2^ = 0.99) gave the same results (data not shown). Both these SNPs showed low to intermediate LD (r^2^≤0.5) with four neighboring markers genotyped, but the possibility that another functional variant lies upstream or downstream and is in LD with SNP s419 or s421 cannot be excluded.

##### IL10RA

In IL10RA only s481 (intron 6) was associated with increased GMT of anti-HBs level and this appeared to be heterozygote protection effect (GMTratio of 1.75, P = 0.001). We observed low LD across the whole length of this gene covered with our set of four SNPs and no LD with neighboring genes tested, so if s481 is in LD with a functional variant it is likely to be very nearby.

Associations of single SNPs in several other genes that had a lesser effect on anti-HBs level, and those taken forward to the 2^nd^ screen are shown in supplementary Table S2; this includes ITGAL (see below and Table 3).

#### ii) anti-HBc status

##### Basic model for anti-HBc status for 1^st^ screen

In the basic model employing the binary outcome measure of core antibody status (see Table 2B) peak anti-HBs level (surrogate for vaccine group and measurement time) clearly affected the risk of core-conversion, with an about 30% increase in being core antibody positive per log decrease of peak anti-HBs level (P = 4.83E-13). The older age groups (>15 years) were more likely to present as anti-HBc positive. A trend towards risk of core-conversion was seen in individuals born in Kantonkunda (excluding the ‘others’ did not change this significantly, data not shown) and to an even lesser extent in males compared with females.

##### Adjusted genetic models for anti-HBc positivity for 1^st^ screen

We identified CD163 as gene to play a role in determining anti-HBc status although polymorphisms in several other genes were also associated with an increased/decreased risk of core-conversion with a reduced size of effect or significance level (see Table 3). The single SNP analysis showed a dose-dependent risk of core-conversion with carriage of the minor allele for CD163 s503 (intron 12); in the multi-SNP analysis this effect seems to be enhanced in the presence of s504 (Ile to Val change at codon 342, r^2^ = 0.56 with s503) with an up to 7- or 4-fold higher risk of core-conversion for those homozygous for the minor allele of s503 and s504, respectively (Table 3). There was no to low LD with markers further upstream in the same gene and its neighbor GNB3, however, there may be further SNPs correlated with s503 and/or s504 downstream in a region not covered in this study.

Inclusion/exclusion of the seven persistent carriers from the anti-HBc positives did not alter the findings for the basic or adjusted genetic models significantly (data not shown).

### Genetic analysis of 2^nd^ screen data

For the second round of genotyping we concentrated on those SNPs that had been shown to increase/decrease the GMT of anti-HBs level in the 1^st^ screen by a factor of 1.5 and/or be associated at the level of P<0.005. Alternative SNPs were genotyped if technical difficulties using the 2^nd^ screen Sequenom genotyping platform were encountered. We also re-screened a few polymorphisms that had shown an effect on anti-HBc status (although the remainder of our samples did not comprise any anti-HBc positive individuals). A total of 50 ‘top’ SNPs were selected, as listed in Supplementary Table S3, of these three failed at the design stage and four failed outright for technical reasons. We obtained good genotyping data for 43 of the ‘top’ SNPs with an average genotyping success rate of 94.8% (ranging from 74.9–99.7%). One SNPs out of these 43 diverged from HWE at the P = 0.05 level.

#### Basic model for peak anti-HBs level for 2^nd^ screen

For the 2^nd^ screen all models including the basic model (i.e. excluding genetic factors, see Table 2A) showed an effect of vaccine group and measurement time on anti-HBs level, as expected and as seen in the 1^st^ screen. Age group, number of doses, gender and village did not appear to influence the vaccine-induced antibody level.

#### Adjusted genetic models for peak anti-HBs for 2^nd^ screen

A summary of the results for the single SNP analysis for the replication study is shown in supplementary Table S4. Out of the total of 43 ‘top’ SNP re-screened we identified one SNP (s595) in ITGAL that appeared to influence peak anti-HBs level. The results for the s595 SNP are comparable in both screens (1^st^ screen: s595_22 GMTratio of 1.71, P = 0.013; 2^nd^ screen: s595_22 GMTratio of 2.15, P = 0.011; Table 3). The s595 polymorphism is a non-synonymous coding change in exon 21 (R791T) and is predicted to affect splicing regulation (FASTSNP prediction). R791T is not in LD with any of the other four screened SNPs in ITGAL (r^2^≤0.1), we thus hypothesize that this is an independent association of a genetic variant that is likely to be functionally relevant.

Other markers screened in the 2^nd^ sample set appeared to increase/decrease the GMT level by more than 1.5-fold, but only showed a trend for association (see supplementary Table S4).

An assessment vaccine non-response (anti-HBs <10IU/ml, primary vaccine failure) was not possible due to lack of power given the small number of non-responders present (N = 47 in 1^st^ and 2^nd^ screen combined). The great majority of study participants were of Mandinka origin and ethnicity was not adjusted for in the analysis. However, inclusion/exclusion of the 26 individuals of other ethnicity from the analysis did not alter significantly the results for the basic or adjusted models for peak anti-HBs level or anti-HBc status (data not shown).

## Discussion

Hepatitis B vaccine-induced immunity is promoted by the presentation of HBsAg via HLA class II molecules on antigen-presenting cells to CD4 T helper cells, thereby triggering HBsAg-specific B cells to proliferate and differentiate into anti-HBs producing cells (primary immune response). Longer term both memory T and B cells are generated, which are responsible for subsequent anti-HBs release in response to later infections (secondary immune responses). Immune memory to HBV is crucially dependent on this rapid antibody release to neutralize the challenge posed by circulating virus. Very little is known about how durable immune memory is maintained, especially in the absence of often undetectable primary vaccine-induced anti-HBs level several years post immunization. However, peak anti-HBs level induced by vaccination is directly related to a uniform decay of antibody level over time irrespectively of population, and predictive of vaccine efficacy long-term[10]. In The Gambia protection against infection was reported to be 83.4% and against persistent carriage to be 96.5% 19 years post vaccination [9]. The present study aimed to investigate whether host genetic factors affect durable vaccine-induced immunity (as predicted by peak anti-HBs level) to HBV infection in The Gambia. An initial screen obtained results on 715 SNPs in 133 genes in 662 infant vaccinees and the replication screen comprised data on 43 SNPs in 393 individuals from the same population.

Based on our basic models, without genetic factors, our overall findings regarding the effect of vaccine group, measurement time, vaccine dose, age group, village and gender were as expected for both our outcome measures and are in line with previous surveys in the West-Kiang region [9]. We saw a strong effect of vaccine group (reflecting vaccine type, regime, route and year of administration) and time elapsed between last vaccine dose and measurement of peak anti-HBs (measurement time) on peak anti-HBs level (Table 2A). None of the other covariates influenced vaccine-induced antibody level substantially.

Individuals positive for anti-HBc despite vaccination in infancy are considered to be core-converters or to have had a ‘breakthrough’ infection. Because all individuals included in our study were considered anti-HBc negative at the time of recruitment, we believe that bias due to infection prior to immunization is not an issue in our analysis. We assessed anti-HBc status in the initial screen only and observed that anti-HBs level (employed as surrogate for vaccine group and measurement time) had the strongest effect on anti-HBc status, whilst gender and village only showed weak associations (Table 2B), as expected. Older individuals appeared more prone to core-conversion, and this higher risk in older age groups could be due to either decreased level of vaccine-induced anti-HBs level or an increased level of exposure through sexual transmission (especially through contact with unvaccinated individuals) or both. Unfortunately we do not have any measure of exposure, other than age as potential indicator of onset of sexual activity, and this could have biased our findings. Due to small numbers present we excluded individuals who had re-converted or presented with variable anti-HBc results, although these would be an interesting sub-group for further investigation.

The HLA region is the most extensively studied area to date with regard to HBV vaccine-induced immunity and relatively consistent data has been reported [29]–[31]. However, reports on other candidate loci are very limited. Polymorphisms in members of the IL1 gene family, IL2, IL4, IL6, IL10, IL12β, TNFα, GNB3, and haptoglobin have all been implicated in affecting the magnitude or kinetics of HBV vaccine-induced antibody response or lymphocyte proliferation [36]–[42]. We concentrated our focus mostly on novel candidate genes outside the MHC region, although our 133 screened genes did include the above mentioned previously screened genes with exception of TNFα and IL2 (see Supplementary Table S1). Our candidate gene selection was based on molecules that are known or likely to influence the regulation of HBV vaccine-induced immune responses such as those involved in pathogen uptake and recognition, antigen processing and presentation, as well as effector and memory T and B cell function (for full list see supplementary Table S1). We hypothesize that host genetic variation could affect increased/decreased peak anti-HBs level, thus indirectly influencing the risk of core conversion, or directly affect susceptibility to infection despite vaccination (as well as potentially re-conversion) and there may be interaction between genes affecting these outcomes (for further details see supplementary Figure S2).

In addition to vaccine group and measurement time (and to a lesser degree village) we found genetic factors to affect our outcomes. Through our 1^st^ screen we identified variation in three candidates (MAPK8, IFNG and IL10RA) that play a role in HBV vaccine-induced immunity by affecting the peak anti-HBs level and polymorphisms in CD163 appeared to affect anti-HBc status (all analyses adjusted for covariates, GMTratio or OR of <0.6 or >1.5 and P≤0.001; Table 3). These and further SNPs in other genes showing reduced size of effect or significance level we followed-up in a 2^nd^ screen (see supplementary Table S2). We did not confirm any previously reported associations in our native African population.

IFNγ is produced by activated T lymphocytes in response to intracellular pathogen challenge. It plays a central role in immune-regulatory processes, e.g. by activating macrophages and potentiating antiviral effects of type I IFNs, and thus is crucial in the cell-mediated immunity. A recent study by Yang *et al* showed that persistent HBV carriers mounted a detectable HBV-specific IFNγ response to a HBV-derived DNA vaccine and this response was observed for at least 40 weeks as a function of CD4+ memory T-cells [43]. Our result suggests that genetically determined differences in IFNγ activity could impact on the magnitude of immune responses to HBV vaccination.

MAPK8 (JNK1) is a kinase which functions within the TLR pathway by promoting the phosphorylation of transcription factors such as c-Jun and ATF2, following stimulation with pro-inflammatory cytokines. A study by Vanlandschoot and colleagues reported reduced phosphorylation of MAPK8 kinase in response to rHBsAg in LPS-stimulated PBMCs [44]. It appears that MAPK8 is primarily involved in the differentiation of T helper into Th1 cells and maintaining their survival during antiviral immune responses [45]. This Th1 mediated favoring of cell-mediated immune responses could be a possible mechanism underlying the association MAPK8 variation with reduced antibody generation following HBV vaccination.

The involvement of IL10RA in immune responses induced by HBV vaccination is also plausible, given that IL10RA is a receptor which mediates the effects of IL10, and IL10 was shown to correlate with immune responses to HBV vaccination [41]. We observed IL10RA variation to have a beneficial effect by leading to increased vaccine-induced anti-HBs level.

The risk of core-conversion despite vaccination was substantially increased in a dose-dependent manner by SNPs in CD163 in our study population. CD163 is a macrophage hemoglobin scavenger receptor involved in acute phase response regulation; it can be induced by IL6, IL10, TLR2 and TLR5, and is suppressed by pro-inflammatory mediators including IFNγ and TNFα. [46]. Reduced levels of IFNγ and TLRs may therefore lead to increased level of CD163 and raised level of CD163 have been reported in patients with persistent hepatitis infection [47]. Our dataset contained seven carriers, but our findings did not change significantly upon inclusion or exclusion of these individuals.

Overall the findings from the replication study did not provide support for the three strongest associations identified in the first screen, perhaps in part as a result of the smaller sample size in the replication study. However, the 2^nd^ screen replicated an association with host genetic variation in ITGAL, a gene which did show association in the 1^st^ screen albeit at a reduced significance level; evidence of correlation with vaccine-induced antibody level was seen for s595. This SNP is a common non-synonymous coding change (R719T; rs2230433; MAF = 38%) and a similar effect size, i.e. an about 2-fold increase in anti-HBs level, was observed in both screens in individuals homozygote for the variant allele encoding for Threonine (1^st^ screen: s595_22 GMTratio of 1.71, P = 0.013; 2^nd^ screen s595_22 GMTratio of 2.15, P = 0.011, see Table 3). Our result for this polymorphism is thus the only true replication in the present study. However, we cannot exclude the possibility of this being a chance finding given the number of tests carried out. Lymphocyte function-associated antigen-1 (LFA1) consists of two subunits ITGAL (also known as LFA1A or CD11a) and ITGB2. ITGAL binds to ICAMs and regulates processes such as leukocyte-endothelial cell interaction, cytotoxic T-cell mediated killing, and antibody dependent killing by granulocytes and monocytes. Despite its central role in immune cell interaction no study assessing responses to viral or vaccine challenges prior to this appears to have assessed host genetic variation in ITGAL. The R791T variant lies within the integrin-alpha-2 domain of the gene, which forms interactions with EGF2, FG-GAP and Integrin_B_tail domains and this SNP is predicted to be involved in splicing regulation, i.e. to play an important role functionally. It would now be of interest to assess this R791T variant for association in an independent sample set of HBV vaccinees or with responses to other vaccines or other diseases that are immunologically mediated.

Lack of power due to small sample numbers prevented us from assessing possible genetic effects on vaccine non-response (primary vaccine failure). Almost 95% of our study population was Mandinka. Ethnicity was not adjusted in our analysis, but inclusion/exclusion of individuals of other ethnic origin did not change any of our results significantly.

There are some limitations to our study: The number of polymorphisms tested in the second sample is small and for technical reasons we could not obtain data for all ‘top’ SNPs identified through the 1^st^ screen. The sample size was about 40% smaller and only one out of our two outcome measures, peak anti-HBs level, could be assessed. The reduced power may explain why our findings from the 2^nd^ screen showed reduced level of significance, although the effect size was comparable for a number of markers followed-up. It is possible that natural boosting through exposure to HBV is a determinant of anti-HBs persistence and we had no means of addressing this specifically in our study. However, a recent vaccine boost study in The Gambia reported on low pre-boost anti-HBs levels suggesting that natural boosting is likely to play a limited role [12].

The strength of our study lies primarily in the availability of good serological and demographic data from infant HBV vaccinees followed up long-term. There is no comparable sample cohort in Africa as far as we are aware. The sole similar study of HBV vaccine efficacy long-term is the Qidong HBV vaccine trial in China, for which data has only been published based on the 5-year follow-up so far [48]. A novel aspect of this present project is the reasonably large number of polymorphisms (715) genotyped across a total of 133 genes located outside the MHC region, the great majority of which have never before been tested as candidate genes in HBV vaccine-induced immunity. We believe it is important to concentrate on novel candidate genes, given that the heritability of non-HLA genes has been estimated to range between 35–50% for immunity induced by HBV vaccination [16], [17].

Findings from studies such as this will help us to increase our understanding of mechanisms underlying vaccine-induced immunity and possible natural immunity to infection. This could lead to the development of genetic markers as correlates of protection. More importantly this type of investigation could help the development of new and improved vaccines, e.g. through use of adjuvants targeting pathways identified through genetic studies, that could eventually lead to life-long protection and reduce the proportion of vaccine failures.

## Supporting Information

Figure S1 Schematic representation of role of host genetic factors in vaccine-induced immunity to HBV. This figure is a schematic diagram of HBV vaccine-induced immunity assessment employing peak anti-HBs level and anti-HBc status as outcome measures. Assuming host genetic variation (here SNP1) correlates with increased/decreased peak anti-HBs level, this in turn indirectly and inversely affects increased/decreased risk of core conversion. To determine whether SNP1 also directly affects anti-HBc status we test for association with anti-HBc status whilst adjusting for anti-HBs level. Simultaneously, there may be other polymorphisms (here SNP2), exerting a direct effect on the risk of core-conversion. There may or may not be interaction between the genes in which these SNPs are located. Additionally, genetic variants modulating the immune-response to HBV vaccination may also affect susceptibility to infection, or other polymorphisms (SNP3) or confounders may lead to a counter effect by increasing the likelihood of infection in the presence of protective vaccine-induced antibody, thus further complicating this scenario. Finally, core antibody positive individuals may over time re-convert, which could be controlled directly or indirectly through genetic factors. In our study only vaccinated individuals with no indication of infection prior to immunization were included, as such there was no scope to assess susceptibility to infection, but simultaneously we believe there was no bias due to infection at the time of recruitment. Furthermore, we excluded individuals who lost anti-HBc positivity or had presented with variable anti-HBc status over the course of follow-up in order to work with a clear-cut distinction of those consistently positive versus consistently negative for anti-HBc.
(0.06 MB TIF)

Figure S2 Outline of study set-up with sample selection and exclusion criteria. For further details see Materials and Methods (study participants).
(0.06 MB TIF)

Table S1 Details for SNPs in 1st screen
(0.16 MB XLS)

Table S2 Single SNP analysis results for 1nd screen for markers taken forward to 2nd screen
(0.04 MB XLS)

Table S3 Details for SNPs in 2nd screen
(0.03 MB XLS)

Table S4 Single SNP analysis results for 2nd screen
(0.03 MB XLS)
