# Correction: Host Genetic Factors and Vaccine-Induced Immunity to Hepatitis B Virus Infection

**DOI:** 10.1371/annotation/0d903d46-1104-43b8-ae6b-4f2343bb3357

**Published:** 2011-02-26

**Authors:** Branwen J. Hennig, Katherine Fielding, John Broxholme, Mathurin Diatta, Maimuna Mendy, Catrin Moore, Andrew J. Pollard, Pura Rayco-Solon, Giorgio Sirugo, Marianne A. van der Sande, Pauline Waight, Hilton C. Whittle, Syed M. Zaman, Adrian V. Hill, Andrew J. Hall

The following information was missing from the Funding section: "The study was supported by funding from the NIHR Oxford Biomedical Research Centre programme."

