# Supplementary figures and images for: Host Genetic Factors and Vaccine-Induced Immunity to Hepatitis B Virus Infection
Source: PLoS One. 2008 Mar 26;3(3):e1898. doi: 10.1371/journal.pone.0001898 (PMC2268746; doi:10.1371/journal.pone.0001898)

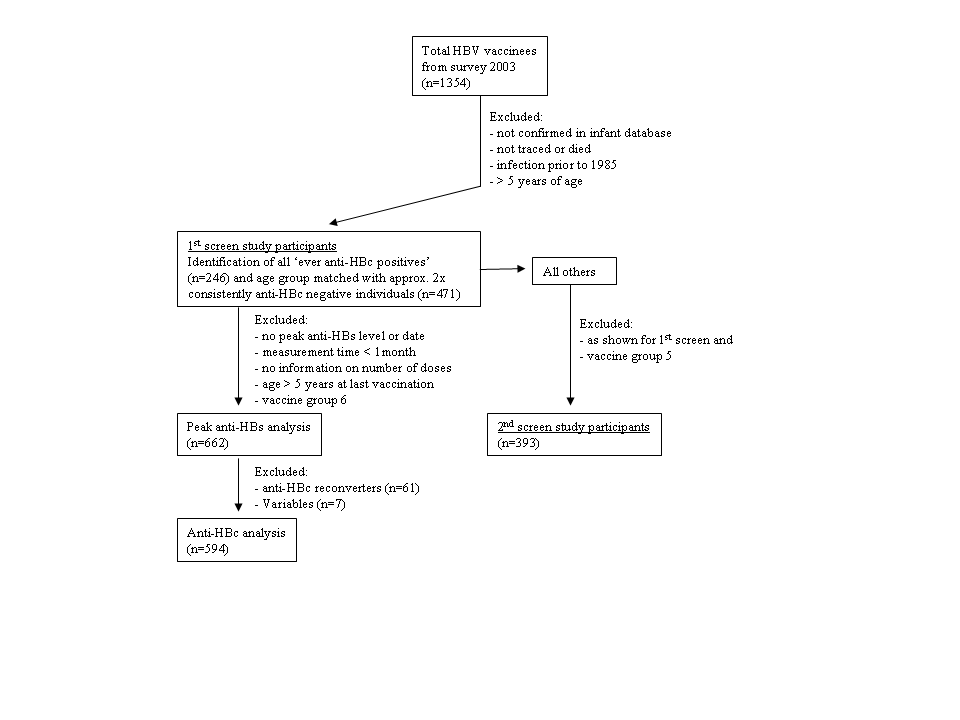

Supplement: Figure S1 — Schematic representation of role of host genetic factors in vaccine-induced immunity to HBV. This figure is a schematic diagram of HBV vaccine-induced immunity assessment employing peak anti-HBs level and anti-HBc status as outcome measures. Assuming host genetic variation (here SNP1) correlates with increased/decreased peak anti-HBs level, this in turn indirectly and inversely affects increased/decreased risk of core conversion. To determine whether SNP1 also directly affects anti-HBc status we test for association with anti-HBc status whilst adjusting for anti-HBs level. Simultaneously, there may be other polymorphisms (here SNP2), exerting a direct effect on the risk of core-conversion. There may or may not be interaction between the genes in which these SNPs are located. Additionally, genetic variants modulating the immune-response to HBV vaccination may also affect susceptibility to infection, or other polymorphisms (SNP3) or confounders may lead to a counter effect by increasing the likelihood of infection in the presence of protective vaccine-induced antibody, thus further complicating this scenario. Finally, core antibody positive individuals may over time re-convert, which could be controlled directly or indirectly through genetic factors. In our study only vaccinated individuals with no indication of infection prior to immunization were included, as such there was no scope to assess susceptibility to infection, but simultaneously we believe there was no bias due to infection at the time of recruitment. Furthermore, we excluded individuals who lost anti-HBc positivity or had presented with variable anti-HBc status over the course of follow-up in order to work with a clear-cut distinction of those consistently positive versus consistently negative for anti-HBc. (0.06 MB TIF) [file pone.0001898.s001.tif]

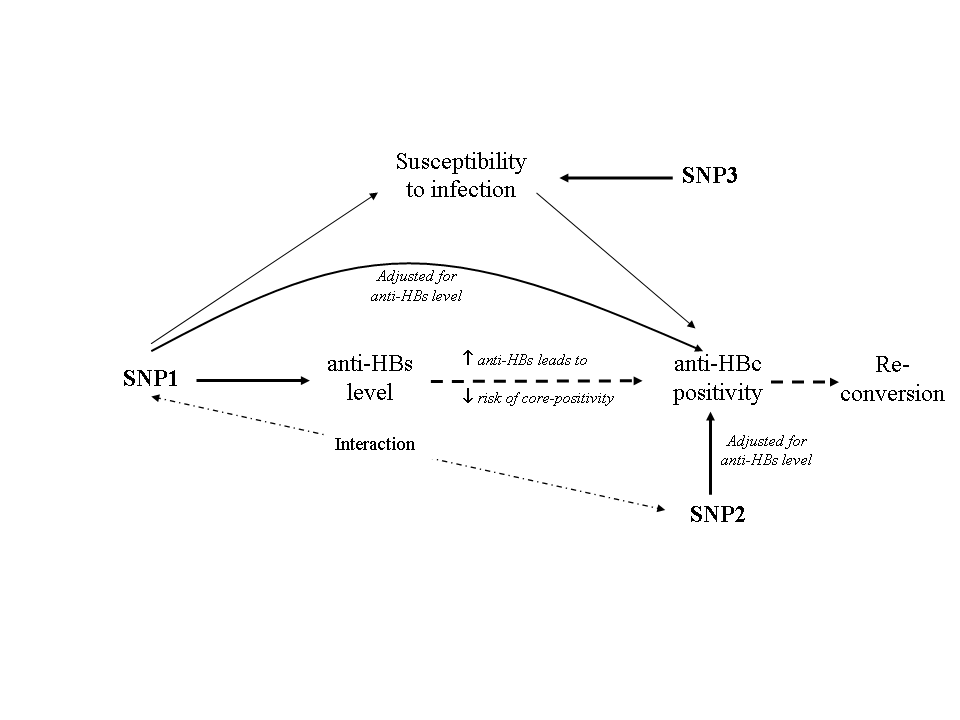

Supplement: Figure S2 — Outline of study set-up with sample selection and exclusion criteria. For further details see Materials and Methods (study participants). (0.06 MB TIF) [file pone.0001898.s002.tif]
